# Convergent Evolution of Neutralizing Antibodies to Staphylococcus aureus γ-Hemolysin C That Recognize an Immunodominant Primary Sequence-Dependent B-Cell Epitope

**DOI:** 10.1128/mBio.00460-20

**Published:** 2020-06-16

**Authors:** David N. Hernandez, Kayan Tam, Bo Shopsin, Emily E. Radke, Karen Law, Timothy Cardozo, Victor J. Torres, Gregg J. Silverman

**Affiliations:** a Division of Rheumatology, Department of Medicine, New York University School of Medicine, New York, New York, USA; b Sackler Institute of Graduate Biomedical Sciences, New York University School of Medicine, New York, New York, USA; c Department of Microbiology, New York University School of Medicine, New York, New York, USA; d Division of Infectious Diseases and Immunology, Department of Medicine, New York University School of Medicine, New York, New York, USA; e Department of Biochemistry and Molecular Pharmacology, New York University School of Medicine, New York, New York, USA; Albert Einstein College of Medicine

**Keywords:** B cell epitope, *Staphylococcus aureus*, antibody function, bacteriophage display, biotechnology, epitope, exotoxins, leukocidin, protection, vaccines

## Abstract

Staphylococcus aureus infection is a major public health threat in part due to the spread of antibiotic resistance and repeated failures to develop a protective vaccine. Infection is associated with production of virulence factors that include exotoxins that attack host barriers and cellular defenses, such as the leukocidin (Luk) family of bicomponent pore-forming toxins. To investigate the structural basis of antibody-mediated functional inactivation of Luk toxins, we generated a panel of murine monoclonal antibodies (MAbs) that neutralize host cell killing by the γ-hemolysin HlgCB. By biopanning these MAbs against a phage-display library of random Luk peptide fragments, we identified a small subregion within the rim domain of HlgC as the epitope for all the MAbs. Within the native holotoxin, this subregion folds into a conserved β-hairpin structure, with exposed key residues, His252 and Tyr253, required for antibody binding. On the basis of the phage-display results and molecular modeling, a 15-amino-acid synthetic peptide representing the minimal epitope on HlgC (HlgC241-255) was designed, and preincubation with this peptide blocked antibody-mediated HIgCB neutralization. Immunization of mice with HlgC241-255 or the homologous LukS246-260 subregion peptide elicited serum antibodies that specifically recognized the native holotoxin subunits. Furthermore, serum IgG from patients who were convalescent for invasive S. aureus infection showed neutralization of HlgCB toxin activity *ex vivo*, which recognized the immunodominant HlgC241-255 peptide and was dependent on His252 and Tyr253 residues. We have thus validated an efficient, rapid, and scalable experimental workflow for identification of immunodominant and immunogenic leukotoxin-neutralizing B-cell epitopes that can be exploited for new S. aureus-protective vaccines and immunotherapies.

## Abstract (precis)

Staphylococcus aureus infection is a major public health threat in part due to the spread of antibiotic resistance and repeated failures to develop a protective vaccine. Infection is associated with production of virulence factors that include exotoxins that attack host barriers and cellular defenses, such as the leukocidin (Luk) family of bicomponent pore-forming toxins. To investigate the structural basis of antibody-mediated functional inactivation of Luk toxins, we generated a panel of murine monoclonal antibodies (MAbs) that neutralize host cell killing by the γ-hemolysin HlgCB.

## INTRODUCTION

Staphylococcus aureus is both a ubiquitous commensal microbe and a leading cause of community-acquired and hospital-acquired bone, joint, lung, and bloodstream infections. Due to the acquisition of broad antibiotic resistance (e.g., in methicillin-resistant S. aureus [MRSA]), this pathogen is increasingly difficult to treat and is now estimated to contribute to more than 20,000 deaths in the United States each year (1). S. aureus infections are also associated with great economic burden, with the cost of treatment of complicated skin and soft tissue infections (SSTIs) alone estimated to be over $5 billion per year for the U.S. health care system (2). Thus, new approaches to prevent and treat S. aureus infections are desperately needed.

In patients, a colonizing S. aureus strain can often be the same as the infecting isolate (3), indicating that the capacity of these isolates to resist host innate immune clearance mechanisms is common but blurring the properties associated with strain pathogenicity. The suppression and evasion of host immunity are mediated by the *in vivo* release of immune cell-targeting S. aureus virulence factors (4, 5). While some of these virulence factors interfere with soluble host factors, such as opsonins and complement proteins, others such as the staphylococcal toxins attack host cellular barriers, red blood cells, and leukocytes. These virulence factors are believed to aid the pathogen in preemptively attacking host defenses. Furthermore, recovery from a serious infection does not uniformly result in persistent augmentation of host protective immunity. S. aureus infection recurrence rates of greater than 20% have been previously reported (6–8), with defects in adaptive immune responses suspected.

Despite many attempts, all efforts to develop an efficacious protective S. aureus vaccine have failed to meet their primary endpoints in clinical trials. At the same time, adults commonly have circulating IgG antibodies (Abs) to hundreds of S. aureus proteins with high reactivity for exotoxins that include the members of the bicomponent pore-forming toxin (PFT) family (9), and memory B-cell responses to PFT members are also common in both healthy adults and those recovering from S. aureus infection (3). These memory responses include antibodies with cross-reactivity between structurally related PFT subunits (3). But little is known about the capacity of such immune responses to affect S. aureus toxin activity, thus leaving open the possibility that neutralizing anti-S. aureus toxin antibodies are important correlates of protective immunity from S. aureus infection just as they are for other pathogens (for example, HIV-1 [10]). Moreover, the characteristics of the epitopes that are targeted by naturally occurring and immunization-induced neutralizing anti-S. aureus antibodies are unknown.

The nine leukocidin members of the PFT family are important contributors to S. aureus strain pathogenic potential (reviewed in reference 11); all share a conserved β-barrel structure (12). The leukocidins are secreted as inactive subunits during infection, but upon binding to the membrane receptors of a targeted host cell, these subunits oligomerize to form pores that act as cell-killing machines that break down epithelial barriers, disable immune cells, and aid the scavenging of nutrients (13, 14). Certain PFTs are associated with specific clinical infection syndromes. For example, the Panton-Valentin leukocidin (LukSF-PV) is associated with primary skin and soft tissue infection and pneumonia (15). Although these factors represent important antigens recognized by host immunity (3, 9), sites within such toxins have been assigned defined functional roles in pathogenesis in only a few cases, such as for the recognition of target cells or at the interface of the assembled toxin subunits required for pore formation (reviewed in reference 11).

HlgC, the γ-hemolysin subunit that belongs to the Luk family of PFTs, is a near-universal component of the core genome of clinical S. aureus isolates (11, 16–18). HlgC binding to the human cell receptor for the chemotactic factor/anaphylatoxin complement fragment C5a (C5aR) occurs at the initiation of the process of intoxication and the death of monocytes and neutrophils that is caused by the holotoxin (19). Furthermore, the introduction of dominant-negative variants or complete ablation of the HlgC subunit inhibits formation of the HlgCB complex *in vitro*, and in S. aureus infection models, elimination of HlgCB has previously been shown to result in dramatic decreases in levels of toxin pore formation and concordant increases in the survival of targeted immune cells (20–23).

As the HlgCB complex is a potent and broadly produced virulence factor responsible for S. aureus subversion of host innate immunity, we sought to characterize the molecular features associated with the recognition of the antigenic determinants by HlgCB-neutralizing monoclonal antibodies (MAbs). We utilized a recently developed filamentous phage-display system that enables the identification of the molecular surfaces recognized by an antibody (24), which we probed with a panel of murine MAbs generated by immunization with the HlgC subunit. From these studies, we identified a minimal toxin subregion that contains a stable β-hairpin loop previously implicated in the functional properties of the toxin. Antibody-dependent toxin neutralization is contingent on the recognition of this subregion and of the critical amino acids therein. Moreover, this small subregion is a common antigenic target of serum antibody responses in human subjects recovering from invasive S. aureus infection, as well as a potent immunogen that elicits murine serum antibody responses that recognize the parental holoprotein.

## RESULTS

### Characterization of the molecular features of the anti-HlgC MAbs.

To investigate the molecular basis for antibody recognition of the HlgC subunit, naive mice were immunized with recombinant HlgC subunit protein, and then immunoassays of postimmune sera were performed to confirm the induction of an anti-HlgC IgG antibody response (data not shown). The spleen from the best responder was then used in a standard hybridoma fusion protocol with sequential subcloning, which resulted in the isolation of four IgG-expressing B-cell hybridomas that displayed high-level reactivity with HlgC. These IgG1-κ monoclonal antibodies (MAbs) were designated anti-HIgC1 MAb, anti-HIgC2 MAb, anti-HIgC3 MAb, and anti-HIgC4 MAb. Each of these four MAbs displayed strong binding activity with the immunizing HlgC recombinant protein as well as high-level cross-reactivity with LukS, a structurally homologous leukocidin subunit that naturally pairs with the LukF subunit to form the Panton-Valentin leukocidin (LukSF) holotoxin (see Fig. S1 in the supplemental material). We did not observe significant reactivity with the other PFT family members, including LukD, LukE, LukF, HlgA, HlgB, LukABC8, LukAB CC30, and α-hemolysin (Hla) (Fig. S1).

10.1128/mBio.00460-20.1 FIG S1 Binding reactivity of anti-HlgC1, anti-HlgC2, anti-HlgC3, and anti-HlgC4 MAbs with all leukocidin subunits. Each of the four MAbs reacted with the HlgC and LukS subunit holoproteins but not with other leukocidin subunits. Purified recombinant leukocidin subunits were coated on ELISA wells in duplicate at 1 μg/ml. ELISA plates were washed and blocked. Binding by the indicated MAbs (at 2 μg/ml) was detected for the purified toxin subunits by ELISA. Green fluorescent protein (GFP) was used as a negative-control protein. Download FIG S1, PDF file, 0.1 MB. Copyright © 2020 Hernandez et al. 2020 Hernandez et al. This content is distributed under the terms of the Creative Commons Attribution 4.0 International license.

Examination of the encoding antibody genes of these MAbs indicated that the members of this panel of anti-HlgC MAbs were assignable to two distinct sets based on the encoding nucleotide sequences (Fig. S2 and S3). The anti-HlgC1, anti-HlgC3, and anti-HlgC4 MAbs express the same somatically generated heavy chain variable region (VH region) (HV1-85/HD1-3*01 or 2–4*01/HJ4*01) gene which was paired with VL (kV8-19*01/kJ5*01) gene rearrangements, but all of these MAbs were nonidentical and differed from one another by a number of replacement mutations (Fig. S2 and S3). By comparison, the anti-HlgC2 MAb expresses a distinct somatically generated VH region (VH2-9*02/HD2-2*01/HJ4*01) and VL region (IGKV1-117*01/IGKJ5*01); as such, this MAb appears to have arisen from an unrelated B-cell clonal origin (Fig. S1 and S2). Notably, while the size of the light chain variable region third complementarity-determining region, LCDR3, is the same in these two clonal sets (i.e., 9 codons), the sizes of the heavy chain CDR3 (HCDR3) in the two presumed clonal sets are very different (i.e., 4 and 14 codons) (Fig. S2). On the basis of their molecular genetic features, we conclude that this panel represented four distinct nonidentical monoclonal antibodies. These MAbs are likely to have arisen from two independent B-cell clonal precursors, with the differences between the presumed clonally related anti-HIgC1, anti-HIgC3, and anti-HIgC4 MAbs likely representing the imprint of somatic diversification events.

10.1128/mBio.00460-20.2 FIG S2 Amino acid sequence of the variable regions of the four anti-HlgC MAbs. The VH and VL regions of anti-HIgC1, anti-HIgC3, and anti-HIgC4 MAbs are related and are designated clone 1. The VH and VL regions for anti-HlgC2 are designated clone 2. The closest germline gene assignments were made using ImMunoGeneTics (IMGT) V-Quest Web-based software (see Materials and Methods) and are indicated in Fig. S3. Red residues are dissimilar from the germline and represent possible somatic replacement mutations. Download FIG S2, PDF file, 0.1 MB. Copyright © 2020 Hernandez et al. 2020 Hernandez et al. This content is distributed under the terms of the Creative Commons Attribution 4.0 International license.

Since these MAbs displayed similar cross-reactivity profiles (Fig. S1), we wondered whether other functional properties of these MAbs are also conserved. Thus, we assessed their capacity for neutralization of the activity of the HlgCB cytotoxicity on primary peripheral blood neutrophils from healthy adults. For these studies, we used a validated *ex vivo* assay (25) and a concentration of HlgCB subunits that kills 90% of cells (24 nM or 0.85 μg/ml). Significantly, each of these four HIgC-reactive MAbs displayed dose-dependent neutralization of HIgCB complex-induced neutrophil intoxication and death, which was highly reproducible using neutrophils from four different donors, with maximal levels of 55% to 84% inhibition for each of the MAbs (data not shown). In contrast, these MAbs showed no detectable neutralizing activity for the LukSF toxin that has a different host cell surface target (data not shown) (reviewed in reference 11).

### Identification of the primary sequence-dependent epitope recognized by the anti-HlgC MAbs.

**FIG 1:**
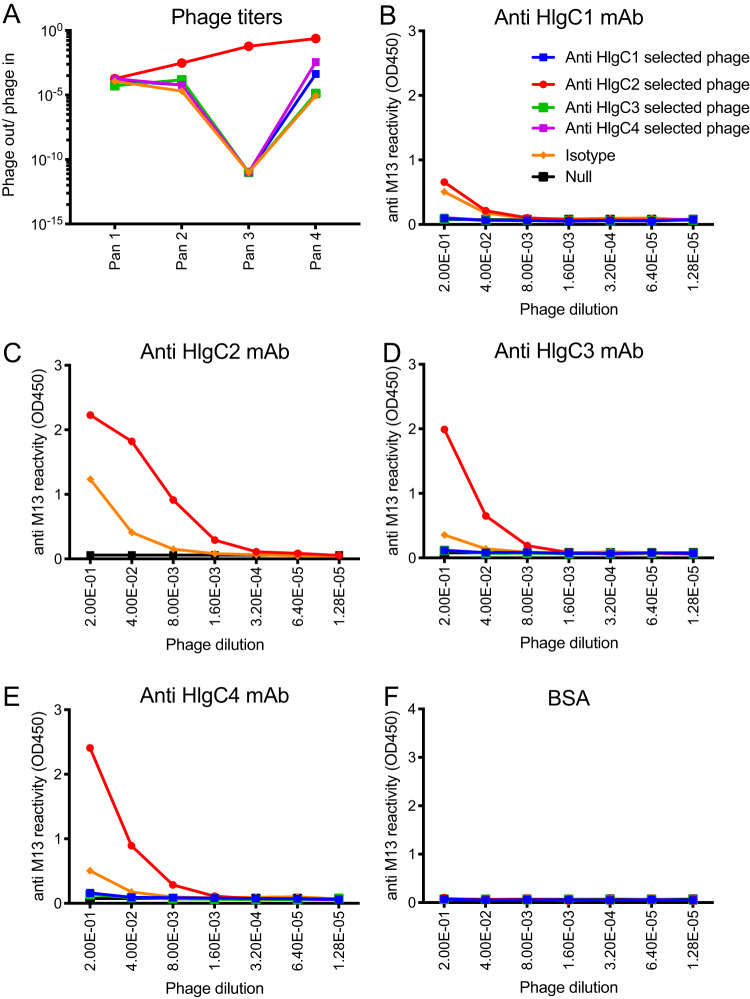
The murine anti-HlgC2 antibody selected for a strongly reactive Luk gene fragment phage sublibrary. (A) Comparisons of the ratios of phage-out/phage-in, from wells of a library subjected to MAb biopanning, demonstrated most-efficient selection by the anti-HlgC2 MAb. After the 3rd and 4th round of biopanning, relatively greater phage expansions were mediated by the anti-HlgC2 MAb. (B to F) Binding of phages from pan 4 to anti-HlgC MAbs. The indicated phages selected by each MAb after pan 4 were put on ELISA plates precoated with (B) anti-HlgC1, (C) anti-HlgC2, (D) anti-HlgC3, (E) anti-HlgC4, and (F) BSA. Binding of phage to the MAbs was detected by the phage-specific anti-M13 antibody. The anti-HlgC2 MAb selected library appeared to be the most strongly reactive with all of the Luk binding MAbs. In these studies, microtiter wells were coated with each of the indicated MAbs or the control protein, BSA, and then equal titers corresponding to the amounts of the indicated anti-HlgC MAb-selected Luk fragment library were added, and phage binding was detected with a labeled anti-M13 antibody. “Isotype” refers to reactivity of wells that had been coated with a murine MAb of irrelevant binding specificity. In these ELISA, each point was assayed in duplicate. OD450, optical density at 450 nm.

To localize the antigenic binding site recognized by these anti-Luk MAbs, we used our recently developed gpVIII-fusion phage-display vector system, pCOMB-Opti8 (24). This phagemid system enables the generation of large libraries of individual phagemid members, each carrying a gene insertion that is expressed as a fusion protein product with a coat protein on the phage surface that provides a physical linkage with the encoding gene (26). Here, we utilized a proven phage-display library composed of random gene fragments of the highly homologous Luk/PFT family member, LukS (24), that was recognized by all of these MAbs (Fig. S1). Using the anti-HIgC2 MAb as bait, we performed four rounds of biopanning, which yielded an increase in the phage-out/phage-in ratio for each of the sequentially selected sublibraries (Fig. 1A) that suggested the successful selection of specific fragment phage clones (24). Furthermore, the pan4 sublibrary selected by the anti-HlgC2 MAb displayed the highest-level dose-dependent binding interactions with the anti-HlgC2 MAb (Fig. 1C). This anti-HIgC2-selected sublibrary exhibited a lower level of reactivity with the anti-HlgC1, anti-HlgC3, and anti-HlgC4 MAbs (Fig. 1B to E), while there was essentially no reactivity with bovine serum albumin (BSA), a control protein (Fig. 1F). Together, these findings suggest that all of the anti-HlgC MAbs displayed similar patterns of reactivity with LukS gene fragment products.

**FIG 2:**
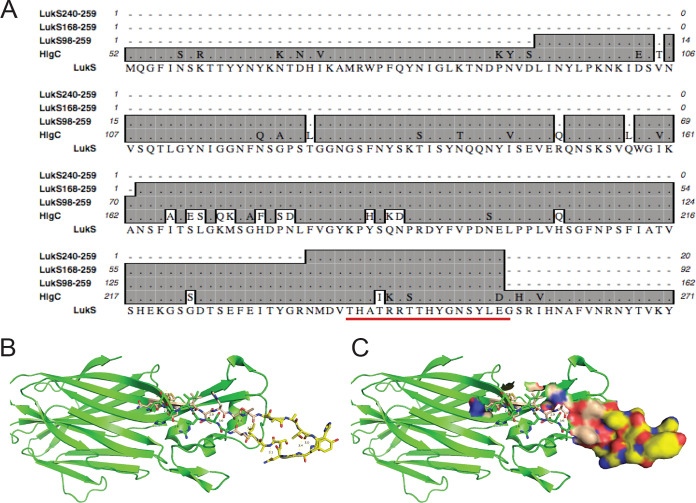
Sequences of phage fragment clones selected by the anti-HlgC2 MAb from the LukS phage display fragment library are nested within the parental gene. (A) Deduced amino acid sequences of recurrent LukS gene fragment phage clones selected by anti-HlgC MAbs (depicted in gray box) align to a conserved region of the PVL LukS homologues, LukS and HlgC. These findings provide a structural basis for MAb cross-reactivity. The LukS244-260 subregion is underlined. (B) Structural visualization of the LukS crystal structure (4IYA) (48), with the conserved solvent-exposed structure among the clones highlighted in yellow with charged surfaces in red and blue. In this solved structure, residues LukS240-242 are not solvent exposed. (C) The solvent-exposed surface of LukS244-260 on the LukS crystal structure is depicted.

To explore the structural basis for antibody binding, 20 colonies were randomly selected from the LukS fragment pan 4 sublibrary recovered by biopanning with the anti-HIgC2 MAb for further analysis. Strikingly, we found that the nucleotide sequences of all of the selected colonies represented only three distinct fragment clones. These LukS fragment clones contained three overlapping LukS gene sequences, ranging from 20 to 162 codons in length, and each was predicted to be in-frame for the potential production of a LukS fragment protein in fusion with the vector gpVIII protein (Fig. 2A).

The anti-HlgC1 and anti-HlgC4 MAbs independently selected for clones that contained only two larger unique in-frame fragments of 92 and 162 codons in length; both included a 20-codon fragment that corresponded to the LukS240-259 subregion. Importantly, this LukS subregion was nearly identical to a homologous subregion in the HlgC gene, with only three conservative amino acid residue substitutions (Fig. 2A) (Table 1). Thus, biopanning of the LukS gene fragment library using phage display and the anti-HlgC MAbs identified a potential common epitope present in both HlgC and LukS (Fig. 2A).

**TABLE 1:** Leukocidin subregions and variants generated as synthetic peptides^*a*^

Leukocidin subregion	Leukocidin variant	Sequence	
LukScyc244-262	biotin-SGSG-C	THATRRTTHYGNSYLEGSR	–C
LukS246-260	biotin SGSG	ATRRTTHYGNSYLEG	
LukScyc246-260	biotin-SGSG-C	ATRRTTHYGNSYLEG	–C
LukScyc248-258	biotin-SGSG-C	RRTTHYGNSYL	–C
LukScyc248-258 mutH252G	biotin-SGSG-C	RRTT**G**YGNSYL	–C
LukScyc248-258 mutY253P	biotin-SGSG-C	RRTTH**P**GNSYL	–C
LukScyc248-258 mutH252G Y253P	biotin-SGSG-C	RRTT**GP**GNSYL	–C

HIgC241-255^*b*^	biotin SGSG	AIKRSTHYGNSYLDG	
HIgCcyc239-257	biotin SGSG-C	THAIKRSTHYGNSYLDGHR	–C

LukScyc182-213	biotin-SGSG-C	NLFVGYKPYSQNPRDYFVPDNELPPLVHSGFN	–C

HIgA123-133	biotin SGSG	YLPKNKIDSAD	

LukScyc189-214	biotin SGSG-C	KPYSQNPRDYFVPDNELPPLVHSGFN	–C

a The parental sequences of Luk subunits LukS and HlgC are depicted in Fig. 2.

b Synthetic peptides were generated with the designated sequence, with an amino-terminal biotin with SGSG linker, or with a carboxyterminal KLH (see Materials and Methods), as specifically described. Residues in bold represent unnatural variants. Residues that differ between the subregions of interest in LukS and HIgC are underlined in the HIgC241-255 peptide.

**FIG 3:**
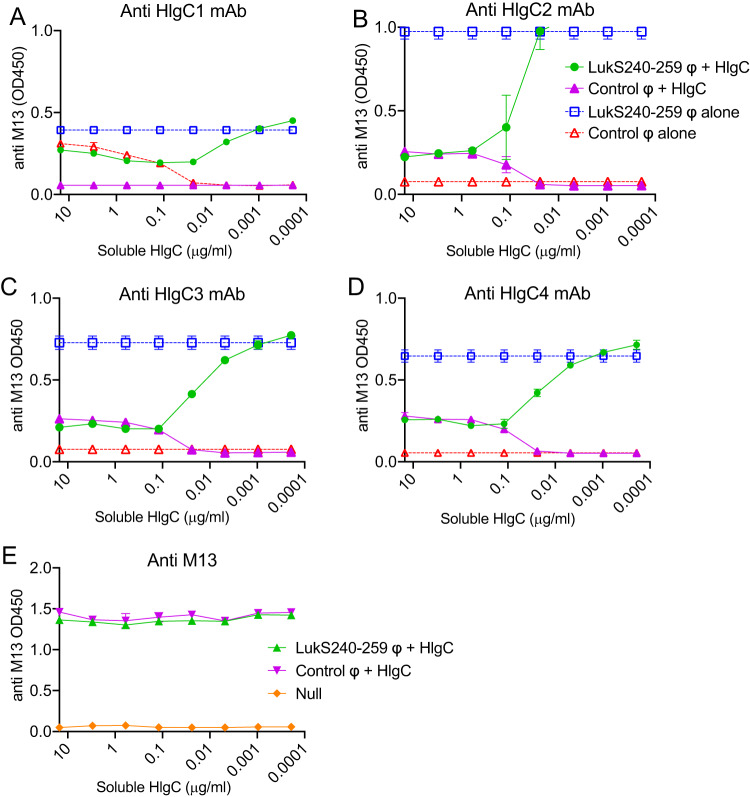
Residues of the LukS240-259 subregion are required for the binding of the anti-HlgC1, anti-HlgC2, anti-HlgC3, and anti-HlgC4 MAbs to the HlgC holoprotein. An individual LukS fragment phage clone, recovered from anti-HlgC MAb pannings, was tested in competition assays. (A to D) Phage particles with surface expression of a LukS240-259 fragment clone competed successfully with the soluble HlgC holoprotein for binding to wells precoated with (A) anti-HlgC1 MAb, (B) anti-HlgC2 MAb, (C) anti-HlgC3 MAb, and (D) anti-HlgC4 MAb in a dose-dependent fashion. Control phage, alone or incubated with soluble HlgC, was not detectable. (E) Equal levels of loading of phage were confirmed by the same assay, and there was direct interaction between phage and the HIgC subunit. In these studies, individual purified phagemid fragment clones were tested in competition with titers representing amounts of the HlgC holoprotein. In these ELISA, representation of phage bound to the precoat was detected with an enzyme-tagged anti-M13 antibody. Results of additional inhibition assays performed with a larger LukS fragment displayed on phage clones are shown in Fig. S4.

To validate the candidate epitopes in the gene fragments recovered by phage display, inhibition studies were performed in which wells were precoated with a fixed concentration of a MAb. Mixtures consisting of a fixed amount of a phage clone with a titer corresponding to the amount of the HlgC holoprotein were then incubated in these microtiter wells, which were subsequently developed with tagged anti-M13 phage detection reagent (Fig. 3). These assays documented that the recombinant HlgC protein mediated dose-dependent inhibition of binding by the fragment clones represented by LukS240-259 (Fig. 3) and LukS168-259 (Fig. S4; see also Fig. S5) corresponding to each of the anti-HlgC MAbs. These results validated that the LukS240-259 and the LukS168-259 fragment clones contained the epitope recognized by the anti-HlgC MAbs.

10.1128/mBio.00460-20.3 FIG S3 Sequence data for the somatically generated CDR3 of anti-HlgC monoclonal antibodies. (A) VH region CDR3. (B) VL region CDR3. For each entry, the individual codon position number is shown with the DNA sequence and deduced amino acid sequence data are listed; the closest germline gene assignments were made using ImMunoGeneTics (IMGT) V-Quest Web-based software (see Materials and Methods). Red residues are dissimilar from the germline and represent possible somatic replacement mutations. Nucleotides without aligned germline gene residues may have arisen from somatic mechanisms for N or P insertion. Download FIG S3, PDF file, 0.1 MB. Copyright © 2020 Hernandez et al. 2020 Hernandez et al. This content is distributed under the terms of the Creative Commons Attribution 4.0 International license.

10.1128/mBio.00460-20.4 FIG S4 Reactivity of anti-HlgC1, anti-HlgC2, anti-HlgC3, and anti-HlgC4 MAb HlgC requires residues represented within the homologue LukS168-259 fragment clone phage. Each fragment clone in phage form was incubated with soluble HlgC and then loaded onto ELISA wells coated with individual HlgC MAbs. To detect an interaction of a fragment clone in phage form, anti-M13 antibody was used. (A to D) Interaction of the fragment clone phage was successfully competed by addition of soluble HIgC holoprotein for binding of the (A) anti-HlgC1 MAb, (B) anti-HlgC2 MAb, (C) anti-HlgC3 MAb, and (D) anti-HlgC1mAb in a dose-dependent manner. The presence of control phage, left untreated or treated by incubation with soluble HlgC, did not result in detectable interaction above baseline. (E) Using wells coated with untagged purified anti-M13 antibody, detection with horseradish peroxidase (HRP)-labeled anti-M13 antibody was used to document equivalent amounts of phage in each sample. Download FIG S4, PDF file, 0.1 MB. Copyright © 2020 Hernandez et al. 2020 Hernandez et al. This content is distributed under the terms of the Creative Commons Attribution 4.0 International license.

10.1128/mBio.00460-20.5 FIG S5 Cross-reactive neutralizing anti-HlgC MAb epitope immunization induces IgG responses to parental S. aureus holotoxins. Immunization of mice with (A) KLH-HlgC241-255 or (B) KLH-LukS246-260 resulted in induction of serum IgG antibodies that bound both immunizing peptides and the parental holoproteins, HlgC and LukS, but not the unrelated tetanus toxoid. Compared to peptides with the parental wild-type Luk subregion sequences, IgG binding was greatly diminished with the replacement mutant LukS248-258HY-GP peptide. Sera were evaluated in a multiplex bead-based assay, with results representing means with SD error bars, starting at 1:100 dilution with 10-fold dilutions. Download FIG S5, PDF file, 0.6 MB. Copyright © 2020 Hernandez et al. 2020 Hernandez et al. This content is distributed under the terms of the Creative Commons Attribution 4.0 International license.

### Structural studies of the HlgC candidate epitope.

Since there is currently no publicly available crystal structure of a HlgC monomer, we chose the LukS crystal structure (PDB identifier [ID] 4IYA), which has 77% protein sequence identity to HlgC, to visualize the potential antigenic surfaces associated with the candidate epitope (Fig. 2B and C). The candidate LukS240-260 subregion in the native toxin subunit structure folds into two antiparallel β strands connected by a small β turn, which forms an ideal hairpin with a solvent-exposed surface (Fig. 2C). Very similar substructures are also present in other members of the leukocidin family, LukE and HlgA (not shown). However, examination of the LukE and HlgA crystal structures indicates that the antiparallel β strands that stabilize the conserved β turn in these particular subunits are comparatively shorter, which may contribute to the lack of binding cross-reactivity of the anti-HIgC MAbs with these subunits (Fig. S1). Furthermore, our modeling studies also suggested that the amino-terminal amino acids of the LukS240-260 subregion (Table 1) are buried in the hydrophobic β sandwich core of holotoxin; thus, these residues are unlikely to directly contribute to binding interactions with the anti-HlgC MAbs.

**FIG 4:**
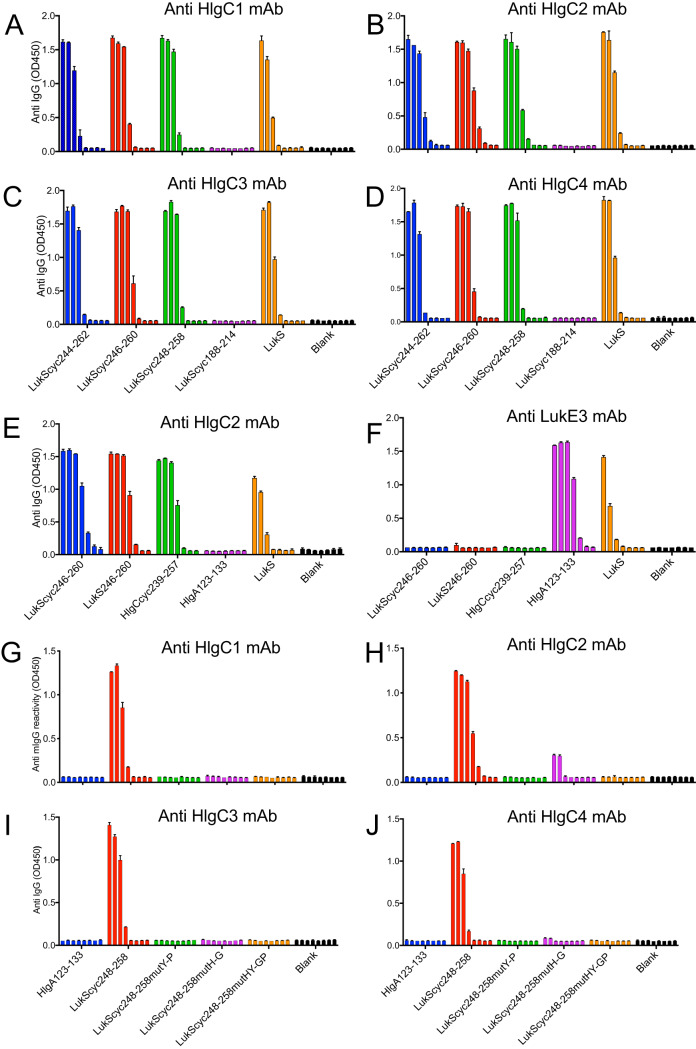
Nested and overlapping minimized LukS peptides are specifically recognized by anti-HlgC MAbs. (A to D) The anti-HlgC1, anti-HlgC2, anti-HlgC3, and anti-HlgC4 antibodies reacted specifically, and in a dose-dependent manner, with LukScyc244-262, LukScyc246-260, and LukScyc248-258 and the LukS holoprotein but not the unrelated LukScyc188-213 peptide. (E) The anti-HlgC2 MAb reacted similarly with the cyclized and noncyclized form of the LukS246-260 subregion and the cyclized form of the homologous HlgC239-257 subregion and the LukS holoprotein but not the unrelated HlgA123-133 subregion. Concurrently, the HlgC241-255 peptide showed comparable levels of dose-dependent reactivity to anti-HlgC2 MAb, which documented the Luk subunit cross-reactivity of this neutralizing antibody. (F) The previously described anti-LukE3 MAb (24) did not recognize the same subregion but did recognize the structurally unrelated HlgA123-133 subregion, which is included here as a binding specificity and isotype control. (G to J) The LukS248-258 His252Gly and Tyr253Pro substitutions (e.g., the autocyclizing double mutant peptide is designated LukScyc 248–259 mutHY-GP) resulted in loss of reactivity with each of the anti-HlgC MAbs, shown individually. These panels show that each anti-HlgC MAb requires the presence of both His247 and Tyr248 to form optimal antibody-peptide complexes. Results represent means with SD error bars for streptavidin-coated microtiter wells, with each biotinylated peptide loaded in a 1:5 dilution series starting at 10,000 ng/ml and detected with each anti-HlgC antibody at 2 μg/ml, performed in duplicate. See Table 1 for peptide nomenclature.

Armed with these insights from the LukS full-length protein, we sought to better localize the minimal LukS epitope recognized by these MAbs. We therefore directly tested the structural requirements for MAb binding and evaluated the possibility of a direct contribution of residues in the β strands. We designed a series of three synthetic peptides with the solvent-exposed subregion, LukS248-258, flanked by various lengths of the antiparallel β strands (Table 1) from the native sequence, which in turn were flanked on both sides by cysteines that formed a disulfide bridge that constrained each peptide into a stable hairpin structure. As all four anti-HlgC MAbs recognized these three synthetic peptides with equivalent levels of reactivity (Fig. 4A to D), the presence of these flanking residues in the β strands did not appear to be critical. These studies suggested that the minimal epitope recognized by all of these MAbs is the LukS248-258 subregion, representing the primary amino acid sequence RRTTHYGNSYL.

On the basis of the findings described above, we then used a similar design to generate a self-cyclizing synthetic peptide that was based on the homologous HlgC subunit, HIgC239-257 (Table 1). As anticipated, side-by-side analyses demonstrated that the peptide with the minimized HlgC239-257 subregion had reactivity equivalent to that seen with the LukS-derived subregion, LukS246-260 (Fig. 4E). The previously described anti-LukE3 MAb (24) does not recognize this same subregion but does recognize the structurally unrelated HlgA123-133 subregion, which is included here as a binding specificity and isotype control (Fig. 4F).

To evaluate whether the intramolecular forces present in the conserved hairpin in the parental protein were sufficient alone to stabilize the fold of the peptide into a conformation recognized by the anti-HlgC2 MAb without the need for covalent cysteine-based cyclization, we also generated a peptide, LukS246-260, devoid of the introduced flanking paired cysteines (Table 1). We found that both constrained and unconstrained LukS246-260 peptides displayed similar strong reactivity with the anti-HlgC2 MAb (Fig. 4E). Collectively, these peptide binding results document that the LukS246-260 and HlgC239-257 subregions are each sufficient as isolated peptides to mimic the surface determinants required for recognition by the anti-HlgC2 MAb.

In the solved crystallographic structure, the His252 and Tyr253 residues are located at the very tip of the β turn of the LukS246-260 hairpin, and the same residues are present at comparable positions in the HlgC241-255 subregion in this homologous Luk subunit. We therefore postulated that residues in the actual β-hairpin loop represented the best candidates for contact sites for binding interactions with one or more of these MAbs. To directly test whether these specific residues were involved in antibody recognition, we designed cysteine-constrained peptides in which either His or Tyr or both in the LukS248-258 core subregion were replaced with Gly or Pro, respectively (i.e., His252Gly and Tyr253Pro) (Table 1). Notably, these substitutions for these Gly and Pro residues were each predicted by molecular modeling to maintain the overall β turn conformation of the native residue. We therefore generated peptides with mutations at position 252 (from His to Gly) and at 253 (from Tyr to Pro) as surface MAb targets because we predicted these would not disrupt the overall epitope architecture. We tested the contribution of these side chains to antibody binding and observed that mutations at either the His residue or the Tyr residue or both did significantly reduce the immune recognition of the LukS248-258 subregion by each of the anti-HlgC1, anti-HlgC2, anti-HlgC3, and anti-HlgC4 MAbs (Fig. 4G to J). Cumulatively, these studies documented that the same epitope (or a closely related epitope) can be emulated by small peptides with the LukS248-258 subregion and the HIgC subregion homologue, where the His and Tyr residues at the tip of the inherently stable hairpin loop represent absolute requirements for the binding interaction of each of the anti-HlgC MAbs.

### The LukS248-258 subregion is required for neutralization of HlgCB toxin-mediated neutrophil killing.

**FIG 5:**
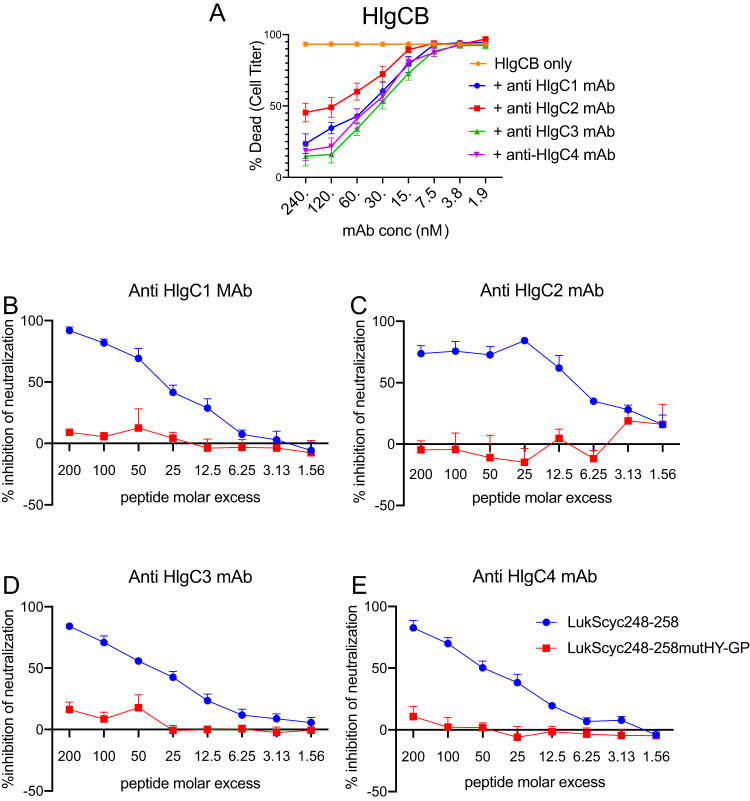
The LukS248-258 subregion is required for anti-HlgC MAb-mediated toxin neutralization. Each of the four MAbs had the capacity to neutralize HIgCB intoxication of primary human neutrophils. (A) Comparison of the dose-dependent neutralization capacities of individual MAbs. (B to E) Each of the MAbs was separately evaluated for LukS248-258 peptide dose-dependent blocking of the capacity of the MAb to neutralize HlgCB toxin activity. The Luk248-258mutHY-GP peptide had no effect on MAb-mediated toxin neutralization. Anti-HlgC MAbs, at a fixed concentration, were tested for their capacity to neutralize HlgCB toxin activity after preincubation of the MAb with sequential concentrations of peptide. In all of these assays, we also assessed the neutralizing capacity of 2-fold serial dilutions of each of the anti-HIgC MAbs. The percentage of inhibition of neutralization of coincubation for each experimental peptide molar excess value was then estimated by interpolation of values from these MAb titration curves. In these ELISA, each point represents the mean for neutrophils from four independent human donors, with standard errors (SE) represented.

We next investigated whether the LukS/HlgC subregion sequence described above, implicated in immune recognition by the anti-HlgC MAbs, also affects the functional capacity of the HlgCB toxin to intoxicate and kill human primary neutrophils. We therefore used a validated *ex vivo* cytotoxicity assay to empirically identify a concentration of the HlgCB holotoxin that killed 90% of the neutrophils (0.85 μg/ml, 24.09 nM). With this HIgCB concentration, we documented that each of the MAbs caused dose-dependent neutralization of HlgCB-mediated neutrophil killing, and we identified the 50% effective concentration (EC_50_) for each MAb in these assays (i.e., that provided 50% reductions in toxin-mediated neutrophil cytotoxicity) (Fig. 5A).

We preincubated the LukS248-258 peptide or the double mutant LukS248-258mutHY-GP peptide with each anti-HlgC MAbs at the EC_50_. Strikingly, the LukS248-258 peptide significantly inhibited the capacity of the MAbs to neutralize the cytotoxicity of HIgCB (Fig. 5B to E). In contrast, the mutant peptide with substitutions for the critical His and Tyr residues (LukS248-258mutHY-GP) did not interfere with the ability of anti-HlgC MAbs to neutralize HlgCB cytotoxicity (Fig. 5B to E). Taken together, these findings indicate that all four of these anti-HlgC MAbs bind an epitope(s) in which the same small hairpin structure appears to be involved in molecular interactions that require the conserved His and Tyr residues. In these experiments, we thereby identified His252 and Tyr253 as residues critical for the binding of anti-HlgC MAbs to the toxins. These findings therefore further validated the primary sequence-dependent structural basis for the cross-reactivity of the anti-HlgC MAbs. Consistent with these data, the homologous HlgC235-255 subregion has been implicated in the binding interaction of the LukS homologous toxin subunit with the host cell receptor (27).

### The HlgC241-255 epitope is commonly recognized by postinfection human serum antibodies.

**FIG 6:**
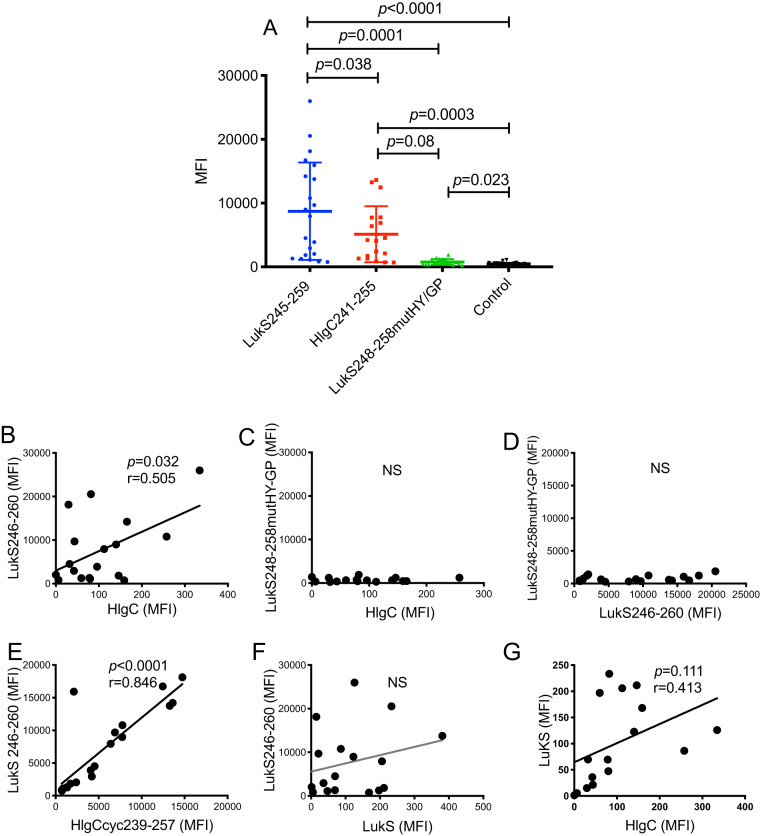
The minimal anti-HlgC241-255 subregion cross-reactive neutralization-associated epitope is commonly recognized by S. aureus convalescent patient sera. (A) Patient serum IgG binding to the LukS246-260 and HlgC241-255 peptides was detected with little or no reactivity to the double replacement mutant control peptide, LukS246-260HY-GP. (B) There was a significant but weak correlation between binding to the LukS246-260 peptide and the HIgC holoprotein, (C) Binding of the HIgC holoprotein did not correlate with binding of LukS248-258 HY-GP double replacement peptide. (D) Binding of the LukS246-260 peptide did not correlate with binding of LukS248-258 HY-GP double replacement peptide. (E) As anticipated, there was a significant strong positive correlation between binding of the homologous LukS246-260 subregion and HlgC241-255 subregion peptides. (F) There was no correlation between binding of the LukS holoprotein and the LukS246-260 subregion. (G) There was no correlation between binding of the LukS holoprotein and the HIgC holoproteins. *n* = 37 convalescent patients with sera obtained at 6 weeks after presentation with S. aureus infection following a full course of antibiotics. Levels of patient serum reactivity for peptides were examined at 1:100 dilution, and binding of holoproteins HlgC and LukS was examined at 1:10,000 dilution, in multiplex bead-based assay. MFI, mean fluorescence intensity; NS, not significant.

To investigate the relevance of the neutralization-associated LukS246-260 and HlgC235-257 minimal epitopes to human immunity, we performed binding assays with these peptides and with sera previously obtained from patients recovering from invasive S. aureus infection (3). In general, the highest titer of S. aureus-specific antibody was detected in convalescent-phase sera collected at about 6 weeks after the initial clinical presentation of the patients (3). In multiplex assays, binding studies were performed with full-length Luk subunit proteins and the isolated minimal LukS246-260 and HlgC235-257 subregion peptides, together with the control LukS248-258mutHY-GP peptide (Fig. 6; see also Fig. S5). Remarkably, the majority of these convalescent-phase sera were enriched for IgG antibodies that recognized the full-length proteins and these minimal epitopes but not the mutant peptides, although the levels of antibody reactivity differed between patients (Fig. 6B to D). Furthermore, the level of IgG reactivity with the LukS246-260 and HlgC239-257 peptides were strongly correlated (*r* = 0.846, *P < *0.0001) (Fig. 6E). The reactivity with the full-length HlgC showed a weak but significant correlation with the recognition of the LukS246-260 related epitope (*r* = 0.505, *P = *0.032) (Fig. 6B). However, the reactivity with the LukS246-260 and the HlgC full-length protein did not correlate with recognition of the peptide LukSmutHY-GP, which contains the sequence mutations that eliminate the Tyr and His residues, which we postulated are critical for antibody binding interactions (Fig. 6C and D). Together, these results suggest that the immune systems of patients, recovering from clinical S. aureus infections, have encountered these toxins and developed antibodies against them, which we postulate leads to the development of IgG antibodies to this conserved hairpin structure in the HlgC and LukS holoproteins.

**FIG 7:**
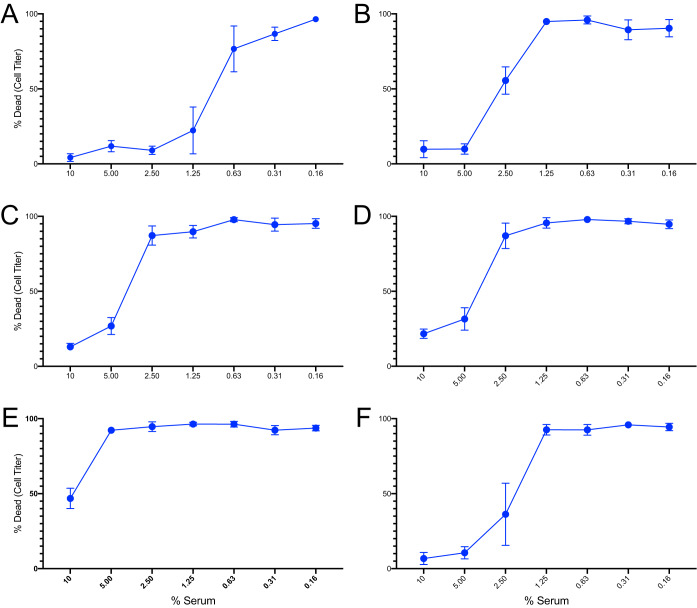
Serum IgG from patients recovering from invasive S. aureus infection neutralizes HlgCB-mediated neutrophil intoxication *ex vivo*. Convalescent patient sera that tested positive for recognition of the peptide were further examined for the capacity of these sera to neutralize HlgCB intoxication on neutrophils. All six patients displayed various degrees of HlgCB neutralization. (A) The serum from patient 1 displayed the greatest range of neutralization with greater than a 50% inhibitory concentration (IC_50_) at 1.25% serum. (B to E) A broad range of neutralization capacity was detected in (B) patient 12 serum, (C) patient 11825 serum, D) patient 53700 serum, and (E) patient 62300 serum that displayed relatively weak neutralization of HlgCB; IC_50_ was observed only at 10% serum. The values presented represent means of results of assays performed using neutrophils from four different healthy donors, with SE shown.

We further sought to test whether convalescent patient sera neutralized HlgCB-mediated cytotoxicity on primary human neutrophils. Indeed, S. aureus postinfection sera were able to inhibit HlgCB intoxication of human neutrophils, and by using a range of serum dilutions, we demonstrated that all of these postinfection serum samples differed in neutralization capacity (Fig. 7).

### HlgC241-255 and LukS246-260 are immunogens that induce *in vivo* responses.

**FIG 8:**
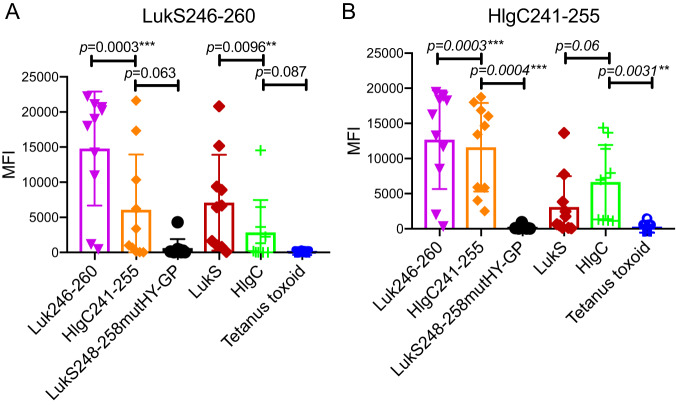
Cross-reactive neutralizing anti-HlgC MAb epitope immunization induces IgG responses to S. aureus holotoxin. Murine immunization with (A) KLH-LukS246-260 or (B) KLH-HlgC241-255 peptide conjugates induced serum antibodies to both LukS246-260 and HlgC241-255, as well as to the holoproteins HlgC and LukS. Notably, reactivity with the replacement mutant LukS248-258HY-GP was greatly diminished compared to the reactivity seen with the peptides with fully native sequences. indicating that Luk conserved residues His252 and Tyr253 contributed to the immunogenicity of this epitope. Reactivity was detected in duplicate with a multiplex bead assay.

For direct testing of the capacity of the HlgC241-255 and LukS246-260 epitopes to induce active immune responses, peptides were synthesized as chemical conjugates with the KLH carrier protein. Following immunization with outbred Swiss Webster mice, the mice mounted IgG antibody responses to the immunizing peptides, which were readily detectable at a dilution of 1:10,000, with cross-reactivity against both peptides but not against the control LukS mutant peptide with His252Gly and Tyr253Pro replacements (Fig. 8). Remarkably, the IgG antibodies induced in these same immunized animals also recognized the HlgC and LukS full-length proteins at levels well above background (Fig. 8). As anticipated, immunization of mice with the HlgC241-255 epitope peptide also induced IgG antibodies with preferential recognition of HIgC compared to LukS (Fig. 8B). Similarly, mice immunized with the LukS246-260 peptide induced an antibody response that displayed greater reactivity with the LukS holoprotein than with HlgC (Fig. 8A). These results suggest that epitope-based immunization induces antibody responses that recognize the parental HlgC and LukS full-length proteins.

## DISCUSSION

S. aureus is a major health threat, and it remains unclear which antigens need to be recognized by the immune system for effective host protection from invasive disease. For our investigations into the antigenic basis of potency and cross-reactivity of antibody against the leukotoxins, we generated four different neutralizing MAbs by immunizing mice with the HlgC toxin subunit. On the basis of the expressed VH:VL gene rearrangements, these antibodies appeared to be derived from two different clonal lineages, and all four antibodies recognized the same or closely related antigenic binding sites, suggesting that these MAbs arose by convergent somatic clonal selection to recognize an immunodominant epitope.

The B-cell epitope(s) of these anti-HlgC MAbs was investigated using a pComb-Opti8 LukS gene fragment phage-display library. This system provided initial evidence identifying the LukS240-259 subregion as the epitope recognized by the MAbs. Indeed, the synthetic peptides, LukS246-260 and the highly homologous HlgC241-255, displayed the same MAb immunoreactivity as the parental protein, HlgC. Furthermore, mutagenesis studies revealed that the binding interactions of the four anti-HlgC MAbs all require the LukS His252 and Tyr253 residues of LukS or the homologous residues in the context of the HIgC subregion. Furthermore, these small subregion peptides compete with the binding of the MAbs with the LukS and HIgC full-length proteins. Interestingly, this epitope contains residues also implicated in host cell receptor binding by the toxin (27), which suggests the molecular basis on which these antibodies neutralize toxin activity.

These Luk subunits, LukS and HlgC, were also recognized by most sera from patients recovering from invasive clinical S. aureus infections. Furthermore, murine immunization with synthetic peptides derived from this epitope induced high titers of IgG response that were cross-reactive with the peptides and the recombinant proteins, LukS and HlgC; these binding interactions were also dependent on the same critical amino acid residues, His 252 and Tyr253 (Fig. 8). Notably, conservation of the same His and Tyr residues in LukS and HlgC is required for immune recognition by the anti-HlgC MAbs, the polyclonal sera from peptide-immunized mice, and serum antibodies from patients recovering from S. aureus infections. These findings indicate that both murine immunity and human immunity involved recognition of the same neutralization-associated B-cell epitope, HlgC239-257/LukS246-260.

The representation of recirculating S. aureus Luk-specific memory B cells does not appear to differ greatly between convalescent patients and healthy adults who presumably have had remote prior S. aureus exposure (3). However, there could be important differences in the associated immune protection linked to binding specificity and neutralization capacity. Since little was known about the structural basis of antibody-mediated HIgC toxin immune recognition and neutralization (23, 28, 29), our study was designed to elucidate the structure-function relationships between the anti-HlgC MAbs and HlgC. Indeed, our results serve to support data presented in our earlier report indicating that human memory anti-Luk responses are commonly cross-reactive between different structurally related Luk PFT subunit members (3). In addition, we now document that such primary sequence-dependent B-cell epitopes are also prominent contributors to antibody-mediated neutralization of HlgCB cytotoxicity for human polymorphonuclear leukocytes (hPMNs). All efforts to develop a protective vaccine have failed to date, and it can also be argued that single-component vaccines may be inadequate to overcome the multitiered assault associated with S. aureus invasive infection. Indeed, the prevention of different staphylococcal clinical infections may require vaccines incorporating different antigens, such as for bacteremia or SSTIs (30, 31), and pairing with an adjuvant that induces a Th1-biased or Th17-biased response may further enhance host protection (32, 33).

Our studies provide further validation of newly developed tools that should be widely applicable for the evaluation of the “fitness” of potential vaccine components of an antistaphylococcal antibody response for recognition of neutralization-associated epitopes. Notably, these validated peptide epitopes can also be used as metrics to guide preclinical reverse vaccinology efforts that seek to mold inducible antibody responses with enhanced protective features against S. aureus infections. In this study, we have identified an immunogenic neutralization B-cell epitope that may be envisioned to have utility as a component in a safe, targeted, multicomponent vaccine. Taken together, our results provide validation for a phage-display-based approach to identify functional minimized Luk PFT epitopes linked to neutralizing antibodies. A future integrated approach may also seek to improve immunization outcomes with minimized epitopes expressed in engineered scaffolds (34), an approach that can potentially stabilize the most desirable conformations of the immunizing peptides. An effective protective vaccine may also utilize a regimen with initial vaccination with full-length holoproteins followed by boosting with a multicomponent formulation of scaffolded neutralizing epitopes for targeting sites that promote toxin neutralization, similar to strategies that are being evaluated for HIV protection (35–37).

Whereas S. aureus infection results in immune exposure to an immense range of protein products, these include a B-cell toxin(s) postulated to interfere with the generation of antigen-specific long-lived plasma cells required for augmentation of host immune defenses (9, 38, 39). To develop an effective protective vaccine, we would focus on efforts designed to enhance vaccine-induced responses directed against minimal epitopes that provide immune protection through neutralization of prominent staphylococcal exotoxins.

In conclusion, we report here the independent recognition of a minimal B-cell epitope by four members of a panel of structurally divergent anti-HlgC monoclonal antibodies. Importantly, the immune systems of humans recovering from invasive S. aureus infections also recognize this immunodominant HlgC241-255/LukS246-260 epitope. Immune recognition of the HlgC241-255/LukS246-260 epitope is dependent on two key residues, His252 and Tyr253, which are conserved in both HlgC and LukS. Finally, we show that the immunodominant epitope in the HlgC241-255/LukS246-260 subregions is immunogenic in mice. Taking the results together, we have identified an important exotoxin determinant that is highly relevant to host defense from infection and that can be exploited in future therapeutic vaccine studies.

## MATERIALS AND METHODS

### Ethics statement.

Animal experiments were reviewed and approved by the Institutional Animal Care and Use Committee of New York University Langone Health (NYULH). All experiments were performed according to NIH guidelines, the Animal Welfare Act, and U.S. federal law.

Leukopaks were obtained from deidentified healthy adult donors with informed consent from the New York Blood Center. Deidentified samples are exempted from the ethics approval requirements by the NYULH Institutional Review Board.

### Generation of murine MAbs reactive with HlgC.

Monoclonal antibodies (MAbs) reactive with the HlgC subunit of the Luk family were generated by immunization of BALB/c mice with recombinant HlgC, using a previously reported standard protocol (24).

### Monoclonal antibody gene sequence determinations.

For determination of antibody gene rearrangements coding for MAbs, we applied a method incorporating rapid amplification of cDNA ends (RACE) along with constant-region primers for unbiased generation of H and κ chain amplimers for Sanger sequencing, as previously reported (24, 40, 41). Alignments to closest germ line genes used the Immunogenetics (IMGT) tool suite (42).

### Neutrophil intoxication and cell lysis assay.

The capacity of a purified recombinant Luk to induce *ex vivo* death of primary human neutrophils was assayed as previously described (25), with primary neutrophils from four healthy donors assessed separately. Unless otherwise indicated, all studies used recombinant HIgCB holotoxin at 0.85 μg/ml (24 nM), which pilot studies demonstrated caused ∼90% cell death as measured by the CellTiter 96 AQueous ONE Solution cell proliferation assay (CellTiter; Promega). Neutralization studies were performed with a MAb or with serum samples at multiple concentrations/dilutions in duplicate. After empirical definition of an effective toxin dose, and of the level of each MAb responsible for 50% toxin neutralization, inhibition studies were repeated under conditions that included individual synthetic peptides with Luk subregion peptides over a range of concentrations.

### Gene fragment library generation and biopanning.

The pComb-Opti8 vector was constructed and a gene fragment library was generated from the gene for the Panton-Valentine S leukocidin subunit (LukS) as described previously (24). Briefly, a pComb3X filamentous phagemid system (43) was modified by use of custom oligonucleotides to introduce a flexible linker and truncated gene VIII optimized for expression of fusion proteins with this major coat protein (44), with the resulting vector termed pComb-Opti8, with gene fragment libraries generated as previously described (24).

### Immunoassays.

Direct binding and competition assays were adapted from earlier described methods (3, 24, 45, 46). Phage binding enzyme-linked immunosorbent assays (ELISA) have been previously described (24).

### Bioinformatic analysis of biopanning of recovered clones.

The sequences of all hits from the phage display were aligned with the whole parent leukotoxin sequence using zero-end gap (ZEGA) global sequence alignment (47). Most hits contained a continuous fragment identical in sequence with a fragment in the hololeukocidin subunit, enabling easy identification and visualization by PyMOL (Schrodinger Inc., New York, NY) of the three-dimensional (3D) structural location of the hit peptide (epitope) within the holoprotein crystallographic structures for LukS (4IYA) (48) and HlgA (4P1Y) (49). These structural locations were then examined for surface exposure or burial to narrow the range of the exact continuous, surface-exposed loop peptide fragments likely to represent the MAb antigenic target.

### Design and synthesis of peptide probes.

For each subregion sequence under analysis, predicted solubility was calculated for the sequences of the exact continuous surface-exposed loop peptide fragments, as recently described (24). When the solubility was acceptable, each sequence was rendered as a full-atom 3D structure computationally and was subjected to *ab initio* folding, which accurately predicts the dynamic 3D conformation of the free peptide as if it were in solution (50–61). *Ab initio* folding used a conformational search algorithm (Biased-Probability Monte Carlo search [62]) to generate all the possible 3D conformations of the peptide. The energy of each conformation, which included thermodynamic and physics-based components, was then calculated (63–66). The lowest energy conformation was thereby identified. In the case of candidate linear B-cell epitopes, this conformation was compared to the *in situ* conformation within the holodomain, using superimposition and contact area algorithms (ICM-Pro software; Molsoft LLC, La Jolla, CA). Modeling studies confirmed that, as isolated peptides, each of these candidate epitopes retained a 3D structure mimicking that in the holotoxin. *Ab initio* folding was also used to evaluate the conformational effects of the specific Gly and Pro mutations in the peptides. To facilitate coating onto neutravidin/streptavidin on beads or on microtiter wells for ELISA, these peptides were then commercially synthesized (InvivoGen) with N-terminal biotin followed by a SerGlySerGly linker adjacent to the PFT subunit subregion of interest (24).

### Murine immunization to assess immunogenicity.

Aliquots of HlgC241-255-KLH or LukS246-260-KLH, were separately emulsified in adjuvant (TiterMax Gold; Sigma). Six-week-old ND4 Swiss Webster outbred female mice were prebled and then primed and subsequently boosted twice at 2-week intervals; the mice were then bled and sera prepared and stored at 80°C until used. IgG antibody responses in each serum were evaluated using a previously described multiplex bead assay (24, 45, 46, 67).

### Statistical analysis.

Data are presented as means ± standard deviations (SD) or as medians and interquartile ranges. The Student unpaired *t* test with Welch correction was used in 2-group comparisons of normally distributed data, whereas the Mann-Whitney nonparametric test was used when the normality assumption was not met. To test for correlations between two variables, the Spearman test was used. *P* values were considered significant at 0.05 for two-tailed tests. Prism software Version 7 (GraphPad) was used for all analyses or as indicated.
