# Supplementary material for: Convergent Evolution of Neutralizing Antibodies to Staphylococcus aureus γ-Hemolysin C That Recognize an Immunodominant Primary Sequence-Dependent B-Cell Epitope
Source: mBio. 2020 Jun 16;11(3):e00460-20. doi: 10.1128/mBio.00460-20 (PMC7298706; doi:10.1128/mBio.00460-20)
Supplement: FIG S1 [file mBio.00460-20-sf001.pdf]

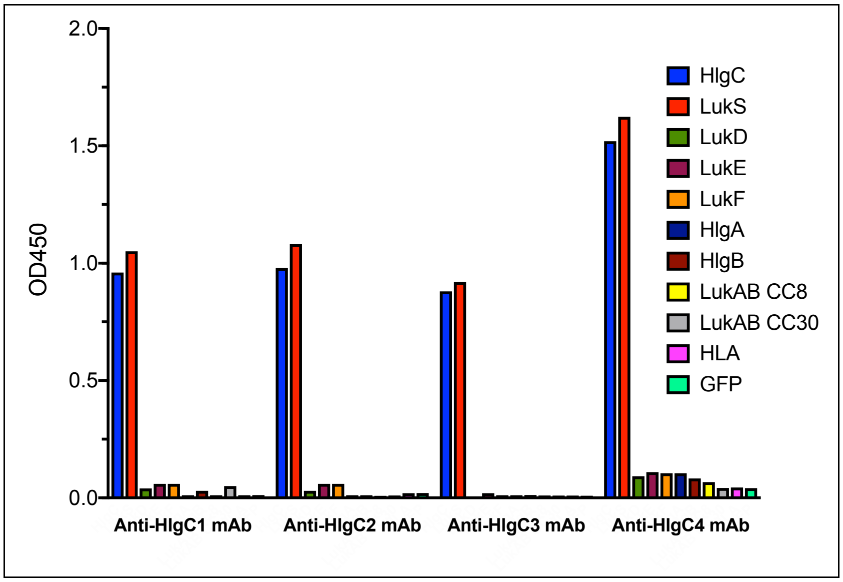

Supplemental Figure 1. Binding reactivity of Anti-HlgC 1, 2, 3 and 4 mAbs with all leukocidin subunits. Each of the four mAbs reacted with the HlgC and LukS subunit holoproteins, but not other leukcodin subunits. Purified recombinant leukococidin subunits were coated on ELISA wells in duplicate at 1 μg/ml. ELISA plates were washed and blocked. Binding was detected by indicated mAbs (at 2 μg/ml) for the purified toxin subunits. by ELISA. GFP is a negative control protein.
