# Supplementary material for: Convergent Evolution of Neutralizing Antibodies to Staphylococcus aureus γ-Hemolysin C That Recognize an Immunodominant Primary Sequence-Dependent B-Cell Epitope
Source: mBio. 2020 Jun 16;11(3):e00460-20. doi: 10.1128/mBio.00460-20 (PMC7298706; doi:10.1128/mBio.00460-20)
Supplement: FIG S2 [file mBio.00460-20-sf002.pdf]

A VH regions of anti-HlgC mAb genes

	FR1	CDR1	FR2	CDR2
HIgC1	QVQLLQSGAELVKPGASVQLSCKAS	GFTFTK**H**D	INWVRQRP**E**QGLEWIGW	IFPGDGNT
HIgC3	QVQLLQSGAELVKPGASVQLSCKAS	GFTFTKYD	INWVRQRPDQGLEWIGW	IFPGDGNT
HIgC4	QVQLLQSGAELVKPGASVQLSCKAS	GFTFTKYD	INWVRQRPDQGLEWIGW	IFPGDGNT
HIgC2	QVQLKESGPGLVAPSQSLSITCTVS	GFSLTNFA	VHWVRQPRGKGLEWLGS	IWADGRT

	FR3	CDR3	FR4
HIgC1	KYN**E**KFKGK**AT**LTTDKSSSTAYMQL**NG**LTSEDSAVYFCA**K**	EIFY	WGQGTSVTVSS
HIgC3	KYNDKFKGKVILTTDKSSSTAYMQL**S**RLTSEDSAVYFCAR	EIFY	WGQGTSVTVS**A**
HIgC4	KYNDKFKGKVILTTDKSSSTAYMQLTRLTSEDSAVYFCAR	EIFY	WGQGTSVTVSS
HIgC2	IYSALMSRLSISKDNSKSQVFLNMNSLQTDDTAMYYCAT	PFYFGYDGGYAMDH	WGQGTSVTVSS

B VL regions of anti-HlgC mAb genes

	FR1	CDR1	FR2	CDR2
HIgC1	DIVMTQSPSSLTVTAGEKVTMSCKSS	QSLLNSENQRNY	LTWYQQKPGQPPKLLIS	WAS
HIgC3	DIVMTQSPSSLTVTAGEKVTMSCKSS	QSL**F**NSENQRNY	LTWYQQKP**R**QPPKLLIS	WAS
HIgC4	DIVMTQSPSSLTVTAGEKVTMSCKSS	QSLLNSENQRNY	LTWYQQKPGQPPKLLIS	WAS
HIgC2	DVLMTQTPLSLPVSLGDQASISCRSS	QSIVHGSGNTY	LEWYLQKPGQSPKLLIY	KVS

	FR3	CDR3	FR4
HIgC1	TRESGVPDRFTGSGSGTDFTLTISSVQAEDLAVYYC	QND**F**SYPLT	FGAGTKLELR
HIgC3	TRES**E**VPDRFTGSGSGTDFTLTISSVQAEDLAVYYC	QNDYSYPLT	FGAGTKLELR
HIgC4	TRESGVPDRFTGSGSGTDFTLTISSVQAEDLAVYYC	QNDYSYPLT	FGAGTKLELR
HIgC2	NRLSGVPDRISGSGSGTDFTLKINRVEAEDLGVYYC	FQGSHVPYT	FGGGTKLEMK

Supplemental Figure 2. Amino acid sequence of the variable regions of the four anti-HlgC mAbs. The VH and VL regions of anti-HIgC 1,3 and 4 mAbs are related and are designated clone 1. The VH and VL region for anti-HlgC2 is designated clone 2. Closest germline gene assignments were made using ImMunoGeneTics (IMGT) V-Quest web-based software (see methods) and are indicated in Supplemental Figure 3. Red residues are dissimilar to the germline and are possible somatic replacement mutations.
