# Supplementary material for: Convergent Evolution of Neutralizing Antibodies to Staphylococcus aureus γ-Hemolysin C That Recognize an Immunodominant Primary Sequence-Dependent B-Cell Epitope
Source: mBio. 2020 Jun 16;11(3):e00460-20. doi: 10.1128/mBio.00460-20 (PMC7298706; doi:10.1128/mBio.00460-20)
Supplement: FIG S3 [file mBio.00460-20-sf003.pdf]

A VH CDR3 of anti-HlgC mAb genes

```
HlgC1        104 105 106 107 115 116 117 118
              C   A   K   E   I   F   Y   W
             tgt gca aaa gag ata ttt tat tgg
IGHV1-85*01  tgt gca a
IGHD1-3*01            aa gag ata
IGHJ4*01                             tat tgg

HlgC3        104 105 106 107 115 116 117 118
              C   A   R   E   I   F   Y   W
             tgt gca aga gag att ttt tat tgg
IGHV1-85*01  tgt gca aaa ga
IGHD1-3*01            aa gag ata
IGHD2-4*01                 g att
IGHJ4*01                             tat tgg

HlgC4        104 105 106 107 115 116 117 118
              C   A   R   E   I   F   Y   W
             tgt gca aga gag att ttt tat tgg
IGHV1-85*01  tgt gca aaa ga
IGHD1-3*01            aa gag ata
IGHD2-4*01                 g att
IGHJ4*01                             tat tgg


HlgC2        104 105 106 107 108 109 110 111 111.1 112.2 112.1 112 113 114 115 116 117 118
              C   A   T   P   F   Y   F   G   Y     D     G     G   Y   A   M   D   H   W
             tgt gcc acc ccg ttc tac ttt ggt tac   gac   ggg   ggc tat gct atg gac cac tgg
IGHV2-9*02   tgt gcc a
IGHD2-2*01                    tc tac ttt ggt tac   gac
IGHJ4*01                                                         c tat gct atg gac cac tgg
```

B VL CDR3 of anti-HlgC mAb genes

```
HlgC1          104 105 106 107 108 109 114 115 116 117 118
                C   Q   N   D   F   S   Y   P   L   T   F
               tgt cag aat gat ttt agt tat cca ctc acg ttc
IGKV8-19*01    tgt cag aat gat tat agt tat cc
IGKJ5*01                                      g ctc acg ttc

HlgC3          104 105 106 107 108 109 114 115 116 117 118
                C   Q   N   D   Y   S   Y   P   L   T   F
               tgt cag aat gat tat agt tat ccg ctc acg ttc
IGKV8-19*01    tgt cag aat gat tat agt tat cc
IGKJ5*01                                     g ctc acg ttc

HlgC4          104 105 106 107 108 109 114 115 116 117 118
                C   Q   N   D   Y   S   Y   P   L   T   F
               tgt cag aat gat tat agt tat ccg ctc acg ttc
IGKV8-19*01    tgt cag aat gat tat agt tat cc
IGKJ5*01                                     g ctc acg ttc


HlgC2          104 105 106 107 108 109 114 115 116 117 118
                C   F   Q   G   S   H   V   P   Y   T   F
               tgc ttt caa ggt tca cat gtt cca tac acg ttc
IGKV1-117*01   tgc ttt caa ggt tca cat gtt cc
IGKJ2*01                                       tac acg ttc
```

Supplemental Figure 3. Sequence data for the somatically generated CDR3 of anti-HIgC monoclonal antibodies. A) Represents the VH region CDR3. B) Represents the VL region CDR3. For each entry the individual codon position number is shown with the DNA sequence and deduced amino acid sequence data listed, with closest germline gene assignments were made using **I**mMunoGeneTics (IMGT) V-Quest web-based software (see methods). Red residues are dissimilar to the germline and are possible somatic replacement mutations. Nucleotides without aligned germline gene residues may have arise from somatic mechanisms for N or P insertion.
