# Supplementary material for: Convergent Evolution of Neutralizing Antibodies to Staphylococcus aureus γ-Hemolysin C That Recognize an Immunodominant Primary Sequence-Dependent B-Cell Epitope
Source: mBio. 2020 Jun 16;11(3):e00460-20. doi: 10.1128/mBio.00460-20 (PMC7298706; doi:10.1128/mBio.00460-20)
Supplement: FIG S4 [file mBio.00460-20-sf004.pdf]

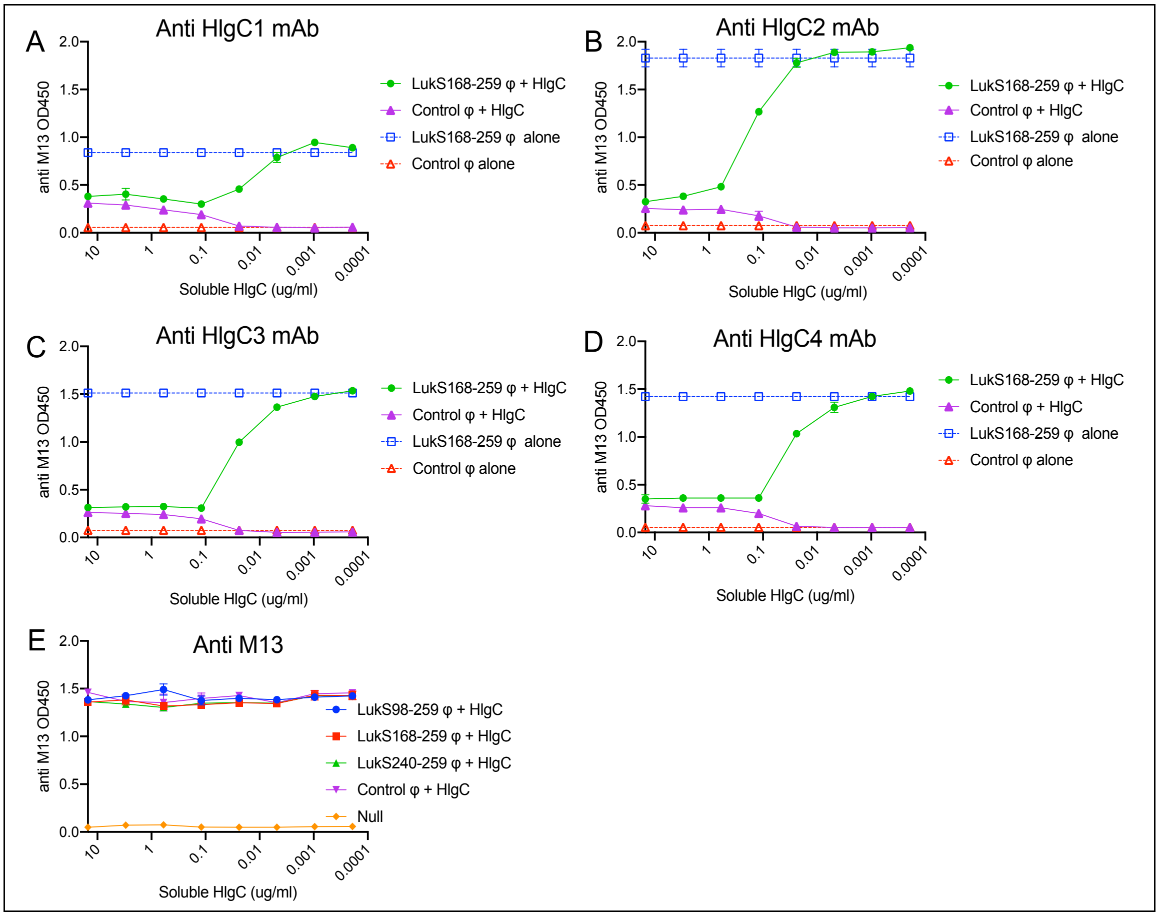

Supplemental Figure 4. The anti-HlgC 1, 2, 3, and 4 mAb HlgC reactivity requires residues the homologue LukS168-259 fragment clone phage. Each fragment clone in phage form was incubated with soluble HlgC then loaded onto ELISA wells coated with individual HlgC mAbs. To detect interaction of a fragment clone in phage form, anti-M13 antibody was used. Interaction of the fragment clone phage was successfully competed by addition of soluble HIgC holoprotein for binding of the **A**) anti-HlgC1 mAb, **B**) anti-HlgC2 mAb, **C**) anti-HlgC3 mAb, and **D**) anti-HlgC1mAb in a dose-dependent manner. Control phage alone, or after incubation with soluble HlgC, did not result in detectable interaction above baseline. **E**) With wells coated with untagged purified anti-M13 antibody, detection with HRP-labeled anti-M13 antibody was used to document equivalent amounts of phage in each sample.
