# Supplementary material for: Convergent Evolution of Neutralizing Antibodies to Staphylococcus aureus γ-Hemolysin C That Recognize an Immunodominant Primary Sequence-Dependent B-Cell Epitope
Source: mBio. 2020 Jun 16;11(3):e00460-20. doi: 10.1128/mBio.00460-20 (PMC7298706; doi:10.1128/mBio.00460-20)
Supplement: FIG S5 [file mBio.00460-20-sf005.pdf]

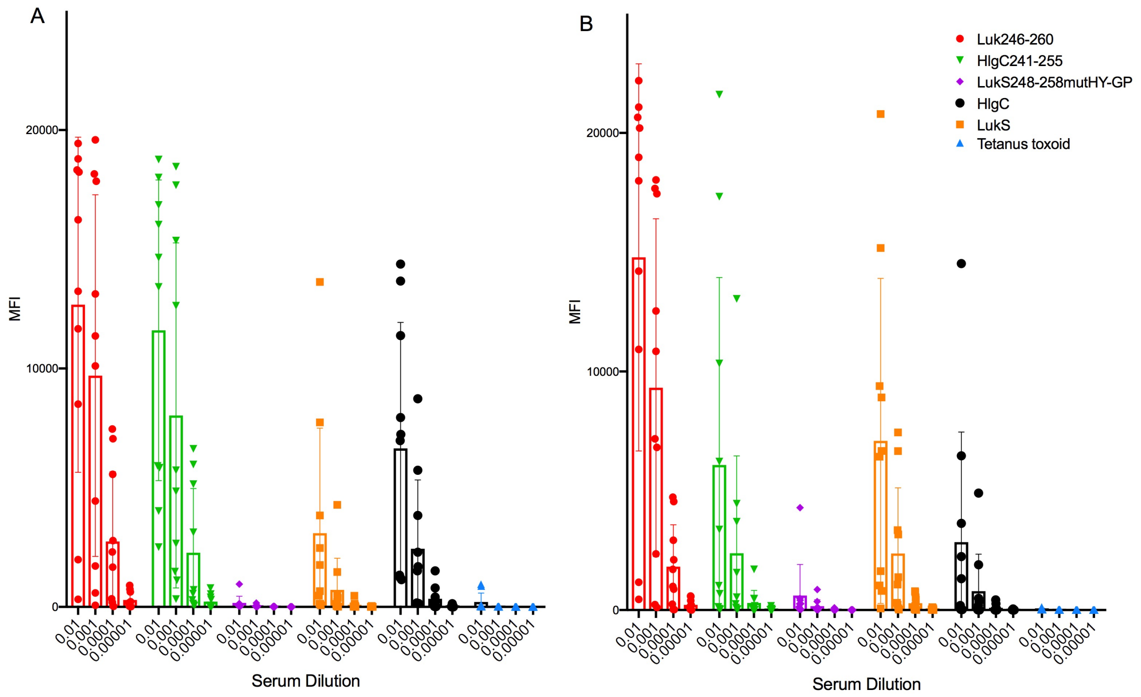

<u>Supplemental Figure 5. Cross-reactive neutralizing anti-HlgC mAb epitope immunization induces IgG responses to parental *Sa* holotoxins.</u> Mice immunized with **A**) KLH-HlgC241-255 or **B**) KLH-LukS246-260 induced serum IgG antibodies that bound both immunizing peptides and the parental holoproteins, HlgC and LukS but not the unrelated tetanus toxoid. Compared to peptides with the parental wildtype Luk subregion sequences, IgG binding was greatly diminished with the double replacement mutant LukS248-258mutHY-GP peptide. Sera were evaluated in a multiplex bead-based assay, with results representing mean with SD error bars, starting at 1:100 dilution with 10-fold dilutions.
